# Effects of tree species mixing on soil microbial community structure in Karst regions of Southwest China

**DOI:** 10.3389/fmicb.2026.1752289

**Published:** 2026-07-30

**Authors:** Yan Wu, Xumeng Tan, Yu Wu, Jianfeng Li, Guili Di

**Affiliations:** 1 College of Biological Sciences, Guizhou Education University, Guiyang, China; 2 Key Laboratory of Biological Resource Development and Utilization in Higher Education Institutions, Education Department of Guizhou Province, Guiyang, China; 3 Key Laboratory of Biological Forest Fire Ecology and Management, Guizhou Provincial Department of Education, Guiyang, China; 4 Industrial Crop Research Institute, Heilongjiang Academy of Agricultural Sciences, Harbin, China

**Keywords:** forest soil, karst, mixed tree species, soil bacterial communities, soil fungal communities, soil microbial community

## Abstract

**Introduction:**

Soil microbial communities are of great significance for maintaining the fragile karst ecosystem. However, research on how tree species configuration drives the assembly of soil microbial communities in this region remains relatively scarce.

**Methods:**

To investigate the effects of tree species mixing on soil microbial community structure in karst regions and its association with environmental variables, this study selected pure *Cryptomeria fortunei*, pure *Liquidambar formosana*, and mixed forests in the Zazuo Experimental Forest Farm of Guizhou Province, China, as research objects. We determined nutrient content of leaves, litter, and soil, as well as soil enzymatic activities. Using high-throughput sequencing technology, we analyzed the diversity and composition of the soil microbial community. Mantel tests and variance partitioning analyses (VPA) were used to elucidate the influence of environmental variables on changes in the soil microbial community.

**Results:**

The results demonstrated that compared with pure *L. formosana* forests, tree species mixing significantly increased the Smith-wilson index of soil bacteria, but decreased the Coverage index of soil fungi (*P* < 0.05). Significant differences in soil fungal β-diversity were observed among different forest types (*P* < 0.05). Besides, the relative abundances of *Actinobacteria* and *Ascomycota* in mixed forests significantly increased by 37.46% and 37.43%, respectively. In contrast, the relative abundance of *Firmicutes* in pure *L. formosana* forests significantly increased by 154.39% compared to that in mixed forests (*P* < 0.05). The relative abundance of *unclassified_p_Ascomycota* in the mixed forest was ~2.81 to 3.39 times that in the pure forests. The relative abundances of *Trichoderma* and *unclassified_c_Agaricomycetes* in the pure *C. fortunei* forests were ~0.35 to 1.88 times those in the pure *L. formosana* forests (*P* < 0.05). Lastly, community composition at the phylum level was significantly correlated with leaf and soil nutrient contents, and soil enzymatic activity. The individual explanatory rates of organic carbon, soil nutrients, and soil enzymes on bacterial community variation were 10.08%, 11.14%, and a negative value, respectively, while synergistic effects between enzymes and organic carbon and between enzymes and soil nutrients explained 39.61% and 32.69%, respectively. For fungal communities, the individual explanatory rates of soil nutrients, soil enzymes, and leaf nutrients were 16.53%, 5.72%, and a negative value, respectively, with synergistic effects between enzymes and leaf nutrients accounting for 0.79%.

**Discussion:**

In conclusion, tree species mixing altered the diversity and composition of soil microbial communities, with environmental variables individually or synergistically influencing microbial community structure. These research findings thoroughly elucidate the effects of stand types on soil microbial community structure in fragile karst ecological areas, and provide a theoretical basis for further improving the functional performance of karst forest ecosystems.

## 1 Introduction

Soil microbes constitute an essential part of soil ecosystems and are the main participants in soil nutrient cycling and energy flow and play a vital role in soil structure improvement, fertility maintenance, environmental pollutant purification, and plant growth (Wu et al., 2022). Changes in soil microbial community diversity and structure can alter soil ecosystem functions and reflect soil health status, thus serving as key indicators for evaluating soil quality and health (Peco et al., 2017). Extensive research has demonstrated that soil properties, including organic matter quality (Li et al., 2022), aggregate structure (Rovira and Greacen, 1957), and available carbon and nitrogen (Yang et al., 2016), along with forest characteristics, including plant diversity (Shang et al., 2021), stand age (Hu et al., 2024), forest density (Yang et al., 2022a; Zarafshar et al., 2024), and stand type (Guo and Gong, 2024; Li et al., 2023; Yang et al., 2022b), influence soil microbial communities. Among these, variation in stand type is considered the primary factor affecting soil microbial diversity and community structure. This was attributed to significant differences in nutrient composition, organic matter content, and root exudate characteristics among stand types, which regulate soil microbial communities through direct and indirect pathways (Guo and Gong, 2024). Furthermore, stand type differences alter microhabitat (for example light, temperature, and humidity), leading to significant divergence in soil microbial community characteristics within the same geographical region (Wang Y. X. et al., 2025).

Stand-type variation, particularly tree species composition, constitutes a focal research area in ecology, particularly concerning its effects on soil microbial communities. The complex regulatory mechanisms exerted by higher litter species richness in mixed forests result in differential effects on soil microbial community structure and diversity (Guo et al., 2024). Positive effects were evidenced by significantly superior vertical spatial distribution and seasonal dynamics of soil microbial abundance following tree species mixing (Zou et al., 2000), effective alleviation of microbial diversity decline through native tree species mixing (Chu et al., 2019), and substantial enhancement of microbial community abundance via introduction of nitrogen-fixing tree species into monocultures by promoting soil microbe-plant root interactions (Dixon and Kahn, 2004). In addition, some studies reveal additional complexities of mixing effects: Li et al. (2021) reported significantly reduced *Ascomycota* abundance in rhizosphere soils of *Pinus massoniana* and *Juniperus formosana* after mixing with broadleaved species, with declining relative abundances of *Saccharomycetes, Pezizomycetes, Leotiomycetes*, and *Xylonomycetes* classes in *P. massoniana* rhizosphere; Yang et al. demonstrated lower *Agaricomycetes* relative abundance in litter but unchanged total fungal abundance in temperate *Larix* mixed forests of Northeast China (Yang et al., 2022b); Guo and Gong (2024) found altered microbial diversity and cross-domain network complexity in conifer-broadleaf mixed forests of Xinjiang's Tianshan arid region, although their community structure resembled monocultures; Li et al. (2023) revealed no enhancement in soil bacterial abundance, diversity and fungal abundance in subtropical *Cunninghamia lanceolata* and *Phoebe bournei* mixtures. These findings indicate significant regional heterogeneity, taxon specificity, and tree species combination dependency in mixed forest effects on soil microorganisms (Guo and Gong, 2024), with driving mechanisms potentially linked to multidimensional variables, including litter properties, root exudates, and soil microhabitats (Li et al., 2023).

Guizhou Province, which harbors the most extensive and intricately developed karst terrain in Southwest China exhibits pronounced ecosystem fragility dictated by its unique eco-geological setting. This fragility manifests as high ecological sensitivity, low environmental carrying capacity, diminished resistance to disturbance, and compromised stability (Zhu, 2003). To remediate degraded ecosystems, China has implemented comprehensive rocky desertification control initiatives across the southwestern karst region. Since the 1990s, successive national afforestation programs, including the Grain for Green Project, Natural Forest Conservation Program, Yangtze River Shelterbelt Initiative, and the Pearl River Shelterbelt Project, which have all been executed systematically. By 2023, the province's forest cover had expanded to 11.1 million hectares, representing 63% of its land area, with planted forests constituting 26% of the total forested landscape (https://lyj.guizhou.gov.cn). Notably, *C. fortunei* and *L. formosana* are the principal afforestation species in this region, accounting for 2.2% and 1.8% of total forest cover, respectively (Zhang and Ding, 2019; Zhang and Guo, 2020). Guizhou Province successfully implemented a *L. formosana-C. fortunei* mixed afforestation model at the Zazuo Experimental Forest Farm, enhancing stand structural complexity. Research on stand type-soil relationships in this region has predominantly focused on water conservation capacity of soil layers (Zeng et al., 2023), soil physicochemical properties and nutrient dynamics (Ni et al., 2017), and soil enzyme activity (Wang Y. X. et al., 2025). However, systematic investigation into how stand type variation, particularly mixed forests, affects soil microbial community characteristics and their driving variables remains a critical knowledge gap.

In summary, the impact of changes in stand type on soil microbial communities has become a focal point in forest soil ecology research. However, existing studies have predominantly concentrated on non-karst regions (Bai et al., 2026; He et al., 2024), with relatively limited reports available on the ecologically fragile karst areas. Furthermore, previous research has often focused separately on the influence of environmental factors on microbial communities (Dangi et al., 2020; Naz et al., 2022), while studies on the synergistic effects of environmental factors driving the assembly of soil microbial communities remain insufficient. Additionally, the mechanisms underlying soil microbial community assembly under different stand types in karst regions are still unclear, leaving the regulatory role of tree species configurations on soil microbial communities incompletely revealed. This study investigated the effects of tree species mixing on soil microbial community diversity and structure in pure *C. fortunei* forests, pure *L. formosana* forests, and their mixed stands at the Zazuo Experimental Forest Farm in Guizhou Province. This study employed Illumina MiSeq high-throughput sequencing technology to investigate the effects of tree species mixing on the diversity and structural characteristics of soil microbial communities and conducted comprehensive analyses of their relationships with key environmental drivers, including foliar nutrients, litter nutrients, soil nutrients, and soil enzymatic activities. The scientific hypotheses addresses were as follows: (1) tree species mixing enhances soil microbial community diversity and structural complexity; (2) tree species mixing modifies leaf nutrient composition, litter nutrient characteristics, soil nutrient content, and soil enzymatic activities; and (3) significant correlations exist between soil microbial community composition and environmental variables (leaf/litter/soil nutrients and soil enzymes). These findings will help elucidate stand type-microbe feedback mechanisms at regional scales and provide microbiology-informed strategies for sustainable management and soil fertility maintenance in native forest ecosystems.

## 2 Materials and methods

### 2.1 Study area description

The study area is situated within the state-owned Zazuo Experimental Forest Farm, Xiuwen County, Guiyang City, Guizhou Province (106°36′-107°3′E, 26°2′-26°59′N). This forested region features a hilly plateau topography and lies within a subtropical monsoon climate zone characterized by abundant precipitation. The area experiences a mean annual temperature of 15.3 °C and mean annual precipitation of 1,129.5 mm, with predominantly acidic to slightly acidic yellow earth soils. The managed area spans 10,108.73 hectares, where dominant tree species include *Pinus massoniana, Cryptomeria fortunei, Pinus armandii, Quercus* spp. and *Cunninghamia lanceolata*. The principal shrubs are *Serissa serissoides, Viburnum dilatatum*, and *Corylus heterophylla*, whereas the herbaceous vegetation is dominated by *Hypolepis punctata, Diplazium esculentum*, and *Ophiopogon bodinieri* (Zeng et al., 2023).

### 2.2 Plot establishment and sample collection

We established three replicate plots for each stand type: pure *C. fortunei* forest, pure *L. formosana* forest, and mixed *C. fortunei-L. formosana* forest within the minimally disturbed areas devoid of significant anthropogenic interference or natural disasters. Replicate plots were spaced >100 m apart, whereas different stand types were separated by >1,000 m (site characteristics are detailed in Table 1). Each 20 m × 20 m (400 m^2^) main plot contained five 2 m × 2 m (4 m^2^) shrub subplots positioned in the southwest, northwest, northeast, southeast, and central locations. Five 1 × 1 m (1 m^2^) herbaceous quadrats were established in each shrub subplot. Representative trees were selected for mature leaf collection using pole pruners and secateurs. Litter samples were collected from herbaceous quadrats. All fresh leaves and litter were labeled, air-dried on newspaper, oven-dried to constant weight at 65 °C, then ground and stored for C, N, P analysis.

**Table 1:** Basic information of the sample plots.

Sample plot number	Forest type	Main tree species	Stand age (years)	Age class	Elevation (m)	Slope (°)	Aspect	Mean tree height (m)	Mean DBH (cm)	Mean height to the first live branch (m)	Tree density (stems/ha)
1	Pure *Cryptomeria fortunei*	*Cryptomeria fortunei*	21	Middle-aged forest	1333	55°	North	17.71	21.72	9.01	625
2			21		1349	45°	Northwest	16.77	23.6	9.96	725
3			23		1323	54°	North	16.54	23.94	9.17	850
4	Pure *Liquidambar formosana*	*Liquidambar formosana*	22	Middle-aged forest	1317	53°	Northwest	18.14	26.81	12.94	650
5			21		1317	48°	Southwest	15.97	27.98	11.62	725
6			21		1317	60.5°	Northwest	16.62	28.25	9.02	750
7	Mixed forests	*Cryptomeria fortunei* and *Liquidambar formosana*	22	Middle-aged forest	1337	58.5°	Northwest	19.32	26.58	9.95	550
8			21		1339	53.1°	Northwest	15.59	30.97	9.69	475
9			22		1350	46°	Northwest	16.06	22.71	8.61	525

Soil samples from the 0–20 cm layer were collected by arranging 5 sampling points according to the “S-shaped sampling pattern”; these samples were mixed into a composite sample. Visible plant and animal residues were removed from the composite sample, which was then passed through a 2-mm aperture soil sieve. The sieved soil was divided into two portions: one portion was stored at −80 °C for high-throughput sequencing of soil microorganisms; the other was spread on newspaper, air-dried to a constant weight, ground, passed through a 0.149-mm aperture sieve, and subsequently used for determination of soil organic carbon (SOC), soil total nitrogen (STN), soil total phosphorus (STP), glomalin-related soil protein (GRSP) content, and soil enzyme activities. Additionally, undisturbed soil samples were collected and soil aggregate fractions were separated using the wet-sieving method, isolating macroaggregates (diameter > 0.25 mm), microaggregates (diameter 0.053–0.25 mm), and silt and clay fractions (diameter < 0.053 mm). These fractions were used to determine organic carbon, total nitrogen, total phosphorus, and glomalin-related soil protein contents within the soil aggregates.

### 2.3 Experimental methods

#### 2.3.1 Determination of leaf, litter, soil nutrient contents, and soil enzyme activities

The organic carbon (OC), total nitrogen (TN), and total phosphorus (TP) contents in leaves, litter, and soil were determined using the potassium dichromate external heating method, semi-micro Kjeldahl method, and molybdenum-antimony anti-colorimetry method, respectively (Bao, 1999). Glomalin-related soil protein (GRSP) content was determined using the Bradford method (Wright et al., 1998). Phosphatase, urease, and catalase activities were determined by disodium phenyl phosphate colorimetry, active phenol-sodium hypochlorite colorimetry, and potassium permanganate titration, respectively (Guan, 1986). Data on leaf litter, soil nutrients, and soil enzyme activity in the study area are presented in Table 2.

**Table 2:** Foliar nutrients, litter, soil nutrients, and soil enzyme activities in different forest stands in the study area.

Category of environmental factors	Environmental factors	LS	FX	HJ
Leaf nutrients	LOC	518.35 ± 4.60	504.64 ± 12.21	497.34 ± 1.55
	LTN	16.07 ± 0.74	15.62 ± 0.95	11.43 ± 0.28
	LTP	1.74 ± 0.05	1.32 ± 0.02	1.43 ± 0.02
Litter nutrients	LLOC	495.27 ± 2.50	467.68 ± 3.13	495.74 ± 3.84
	LLTN	11.34 ± 0.43	12.05 ± 0.58	10.09 ± 0.18
	LLTP	0.79 ± 0.02	0.81 ± 0.01	0.75 ± 0.01
Soil nutrients	SOC	26.43 ± 3.01	23.27 ± 1.56	30.27 ± 1.48
	STN	1.19 ± 0.03	1.03 ± 0.02	1.18 ± 0.08
	STP	0.44 ± 0.01	0.40 ± 0.02	0.41 ± 0.02
	SMSOC	18.59 ± 1.67	22.93 ± 2.91	28.92 ± 4.79
	SISOC	18.37 ± 1.29	21.57 ± 3.63	25.40 ± 4.93
	SCSOC	15.92 ± 1.93	21.01 ± 3.82	18.92 ± 4.93
	SMTP	0.86 ± 0.08	0.60 ± 0.05	0.89 ± 0.02
	SITP	0.76 ± 0.11	0.73 ± 0.04	0.70 ± 0.05
	SCTP	0.86 ± 0.07	0.73 ± 0.05	0.73 ± 0.06
	SMTN	0.19 ± 0.02	0.18 ± 0.02	0.27 ± 0.02
	SITN	0.24 ± 0.03	0.23 ± 0.00	0.14 ± 0.03
	SCTN	0.17 ± 0.02	0.25 ± 0.02	0.08 ± 0.02
	EEG	1.21 ± 0.07	1.34 ± 0.07	1.2 ± 0.22
	SMEEG	1.08 ± 0.16	0.99 ± 0.07	1.03 ± 0.07
	SIEEG	0.85 ± 0.09	0.87 ± 0.04	0.89 ± 0.07
	SCEEG	1.20 ± 0.09	0.95 ± 0.05	1.12 ± 0.02
	TG	5.73 ± 0.47	6.07 ± 0.68	5.77 ± 0.80
	SMTG	4.45 ± 0.32	4.7 ± 0.3	4.41 ± 0.19
	SITG	4.32 ± 0.19	4.42 ± 0.51	3.80 ± 0.37
	SCTG	4.73 ± 0.15	4.69 ± 0.49	4.30 ± 0.30
Enzyme activity	ACP	1.22 ± 0.06	1.17 ± 0.1	0.81 ± 0.28
	URE	4.94 ± 2.25	5.03 ± 1.81	4.34 ± 1.35
	CAT	0.43 ± 0.12	0.56 ± 0.11	0.54 ± 0.05

LS, pure *Cryptomeria fortunei*; FX, pure *Liquidambar formosana*; HJ, mixed forests; LOC, leaf organic carbon; LTN, leaf total nitrogen; LTP, leaf total phosphorus; LLOC, litter layer organic carbon; LLTN, litter layer total nitrogen; LLTP, litter layer total phosphorus; SOC, soil organic carbon; STN, soil total nitrogen; STP, soil total phosphorus; SMSOC, soil macroaggregate organic carbon; SISOC, soil intra-macroaggregate organic carbon; SCSOC, soil-clay organic carbon; SMTP, soil macroaggregate total phosphorus; SITP, soil intra-macroaggregate total phosphorus; SCTP, soil-clay total phosphorus; SMTN, soil macroaggregate total nitrogen; SITN, soil intra-macroaggregate total nitrogen; SCTN, soil-clay total nitrogen; EEG, soil easily-extractable glomalin-related soil protein; SMEEG, soil macroaggregate easily-extractable glomalin-related soil protein; SIEEG, soil intra-macroaggregate easily-extractable glomalin-related soil protein; SCEEG, soil-clay easily-extractable glomalin-related soil protein; TG, soil total glomalin-related soil protein; SMTG, soil macroaggregate total glomalin-related soil protein; SITG, soil intra-macroaggregate total glomalin-related soil protein; SCTG, soil-clay total glomalin-related soil protein; ACP, acid phosphatase; URE, urease; CAT, catalase.

#### 2.3.2 Soil microbial illumina-MiSeq High-throughput sequencing

Soil samples were sent to Shanghai Majorbio Bio-Pharm Technology Co., Ltd. Total genomic DNA of microbial communities was extracted using the DNeasy^®^ PowerSoil^®^ Pro Kit, with DNA quality detected by 1% agarose gel electrophoresis and DNA concentration and purity measured by NanoDrop2000. Using the extracted DNA as a template, the V3-V4 hypervariable region of the bacterial 16S rRNA gene was PCR-amplified with the barcoded forward primer 338F and reverse primer 806R (Liu et al., 2016), and the ITS1 region of the fungal ITS gene was PCR-amplified with the barcoded forward primer ITS1 and reverse primer ITS2R (Adams et al., 2013). The amplification program was: 95 °C pre-denaturation for 3 min; 35 cycles (bacteria) or 27 cycles (fungi) of 95 °C denaturation for 30 s, 55 °C annealing for 30 s, 72 °C extension for 45 s; final extension at 72 °C for 10 min; hold at 4 °C (PCR instrument: ABI GeneAmp^®^ 9700).

Three replicates per sample were processed and PCR products from the same sample were pooled and recovered using 2% agarose gel electrophoresis. Purification was performed using the AxyPrep DNA Gel Extraction Kit, with recovered products verified by 2% agarose gel electrophoresis and quantified using the Quantus™ Fluorometer (Promega, USA).

Purified PCR products were used to construct libraries using a NEXTFLEX Rapid DNA-Seq Kit. Sequencing was performed using the Illumina MiSeq PE300 platform (Shanghai Majorbio Bio-Pharm Technology Co., Ltd.). Raw data were deposited in the NCBI SRA database. Paired-end raw sequences were quality-controlled using fastp (Chen et al., 2018; https://github.com/OpenGene/fastp, version 0.19.6), assembled using FLASH (Magoč and Salzberg, 2011) (http://www.cbcb.umd.edu/software/flash, version 1.2.11), and clustered into operational taxonomic units (OTUs) at a 97% similarity threshold, with chimeras removed using UPARSE (Edgar, 2013; Stackebrandt and Goebel, 1994) (http://drive5.com/uparse/, version 7.1). We selected the 97% identity threshold with targeted rationale: first, our study focuses on overall community structure and taxonomic variations at the genus and higher taxonomic levels, for which 97% OTU clustering provides sufficient resolution to capture core community patterns. Second, this threshold maintains methodological consistency with most prior studies in the same habitat, enabling reliable cross-study comparisons. Third, 97% clustering effectively reduces interference from low-abundance sequencing errors and spurious sequences, yielding more robust results for community-level analyses.

#### 2.3.3 Data analyses

The effects of different forest stand types on leaf, litter, and soil nutrients, and enzyme activities were examined using a one-way ANOVA in SPSS 27.0. Soil bacterial and fungal α-diversity was analyzed using Mothur 1.30.2, calculating community richness (Ace), community diversity (Simpson), community coverage (Coverage), and community evenness (Smith-wilson); intergroup differences were tested using one-way ANOVA. Non-metric multidimensional scaling (NMDS) analysis based on the binary Jaccard distance algorithm was performed to visualize differences in soil microbial community structure among forest stand types. Metastat analysis was used to compare differences in phylum-or genus-level relative abundances among forest types, and FDR correction was performed. The Mantel test examined correlations between soil microbial community composition and environmental variables to quantify the influence of environmental variables and their relationships. Environmental variables significantly correlated with microbial community composition, and variables with high multicollinearity were excluded using Variance Inflation Factor (VIF) analysis. Variance Partitioning Analysis (VPA) was then applied to the categorized environmental variables to determine the relative contributions of the different factor categories to soil microbial community composition. All obtained raw sequence datasets have been uploaded to the NCBI Sequence Read Archive (SRA) with accession number PRJNA1482.

## 3 Results and analysis

### 3.1 Effects of stand types on microbial community diversity

#### 3.1.1 Alpha diversity analysis of bacterial and fungal communities

**Figure 1:**
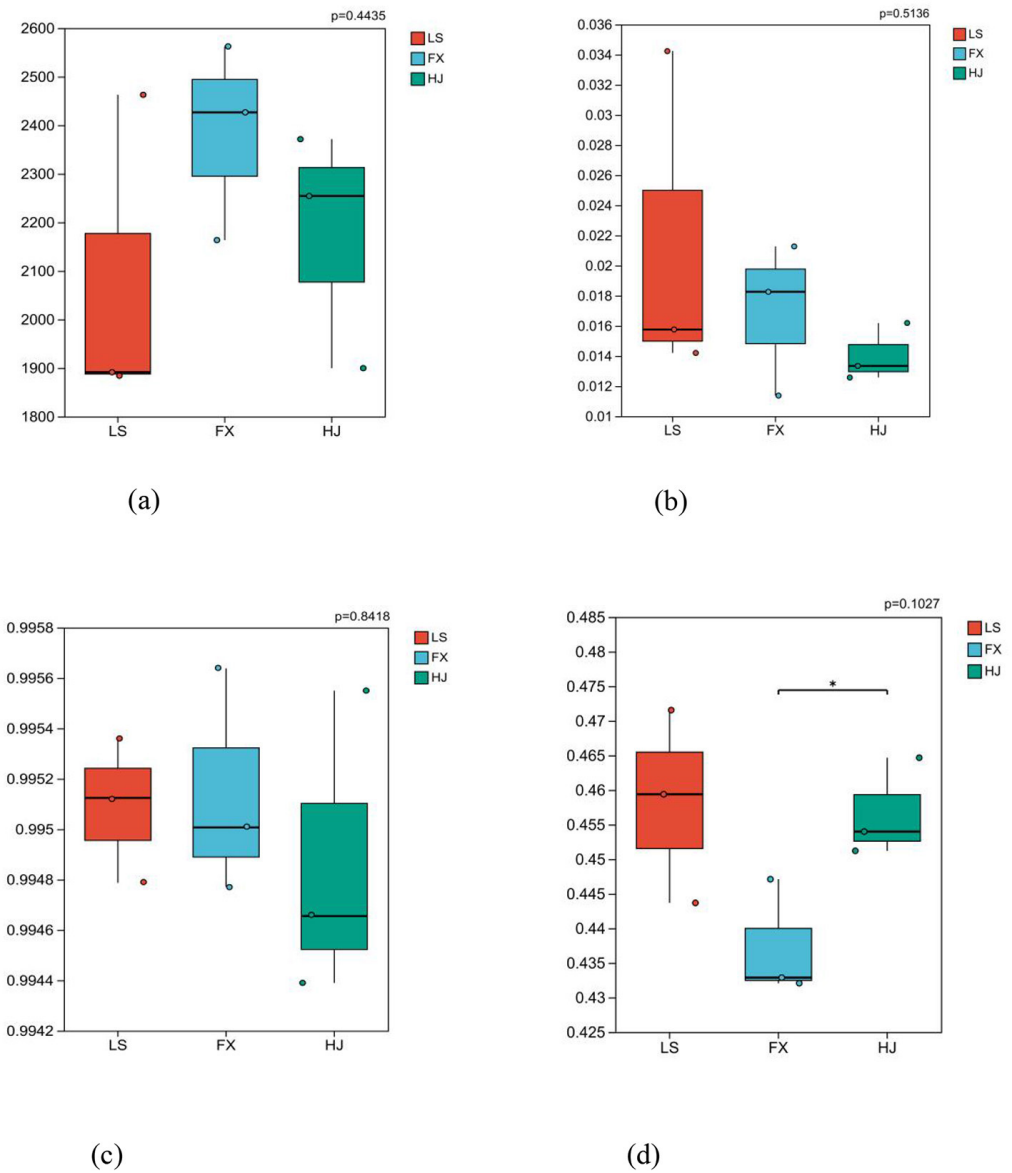
Soil bacterial alpha diversity in different forest stand types. LS, pure *Cryptomeria fortunei*; FX, pure *Liquidambar formosana*; HJ, mixed forests. **(a)** Ace index. **(b)** Simpson index. **(c)** Coverage index. **(d)** Smith-Wilson index.

**Figure 2:**
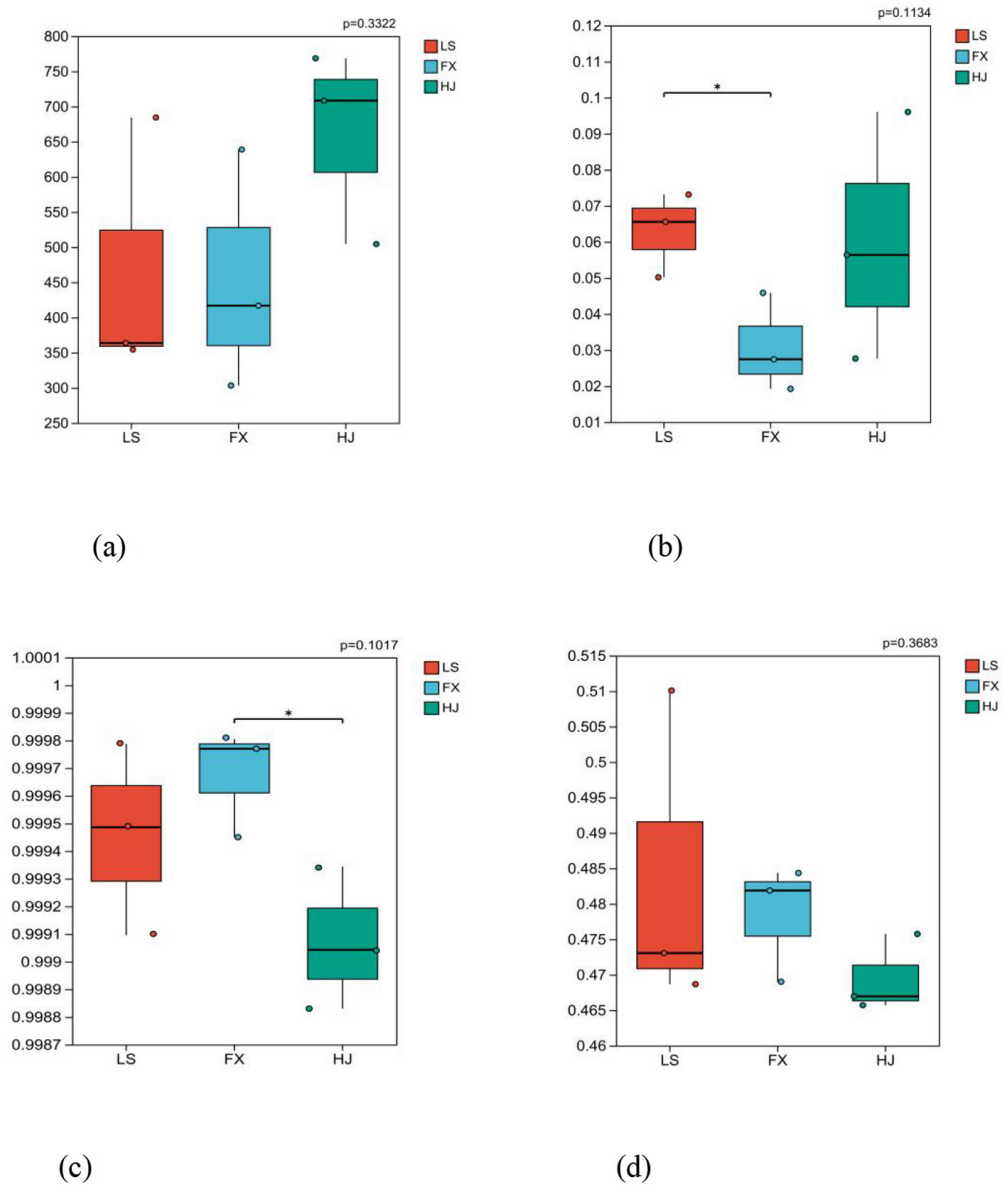
Soil fungal alpha diversity in different forest stand types. LS, pure *Cryptomeria fortunei*; FX, pure *Liquidambar formosana*; HJ, mixed forests. **(a)** Ace index. **(b)** Simpson index. **(c)** Coverage index. **(d)** Smith-Wilson index.

The ranking of soil bacterial Smith-wilson and fungal Simpson indices across stand types were as follows: pure *C. fortunei* forest > *conifer-broadleaf* mixed forest > pure *L. formosana* forest. The bacterial Smith index differed significantly between mixed and pure *L. formosana* forests (1.04-fold greater, *P* < 0.05), whereas the fungal Simpson index showed significant differences between pure *C. fortunei* and pure *L. formosana* forests (2.04-fold greater, *P* < 0.05). Fungal Coverage indices were as follows: pure *L. formosana* forest > pure *C. fortunei* forest > mixed forest, with significantly higher values in pure *L. formosana* than in mixed forests (*P* < 0.05). In contrast, bacterial and fungal Ace indices, bacterial Simpson and Coverage indices, and fungal Smith-wilson index showed no significant differences among stand types (*P* > 0.05). Both bacterial and fungal Coverage indices exceeded 0.99, confirming an adequate sequencing depth for community representation (Figures 1, 2).

#### 3.1.2 Beta diversity analysis of bacterial and fungal communities

**Figure 3:**
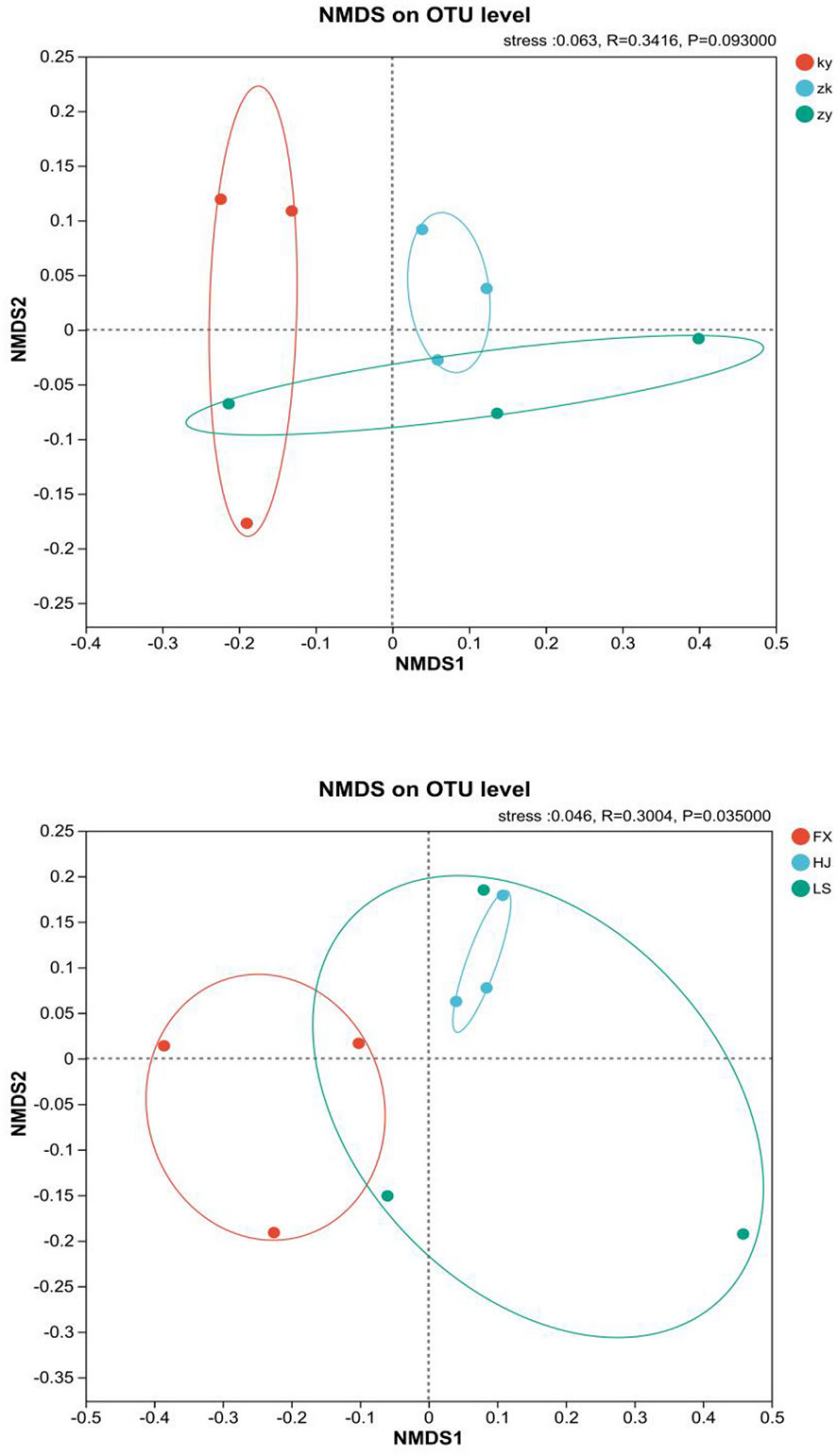
Soil Microbial Beta Diversity in Different Forest Stand Types (Top: Soil Bacteria; Bottom: Soil Fungi). LS, pure *Cryptomeria fortunei*; FX, pure *Liquidambar formosana*; HJ, mixed forests.

NMDS ordination coupled with ANOSIM tests revealed no significant differences in soil bacterial community structure among stand types (*P* > 0.05), whereas fungal communities exhibited significant structural divergence (*P* < 0.05) (Figure 3). Both ordinations demonstrated meaningful configurations with stress values < 0.1, confirming an acceptable ordination quality.

### 3.2 Effects of stand types on microbial community composition

**Figure 4:**
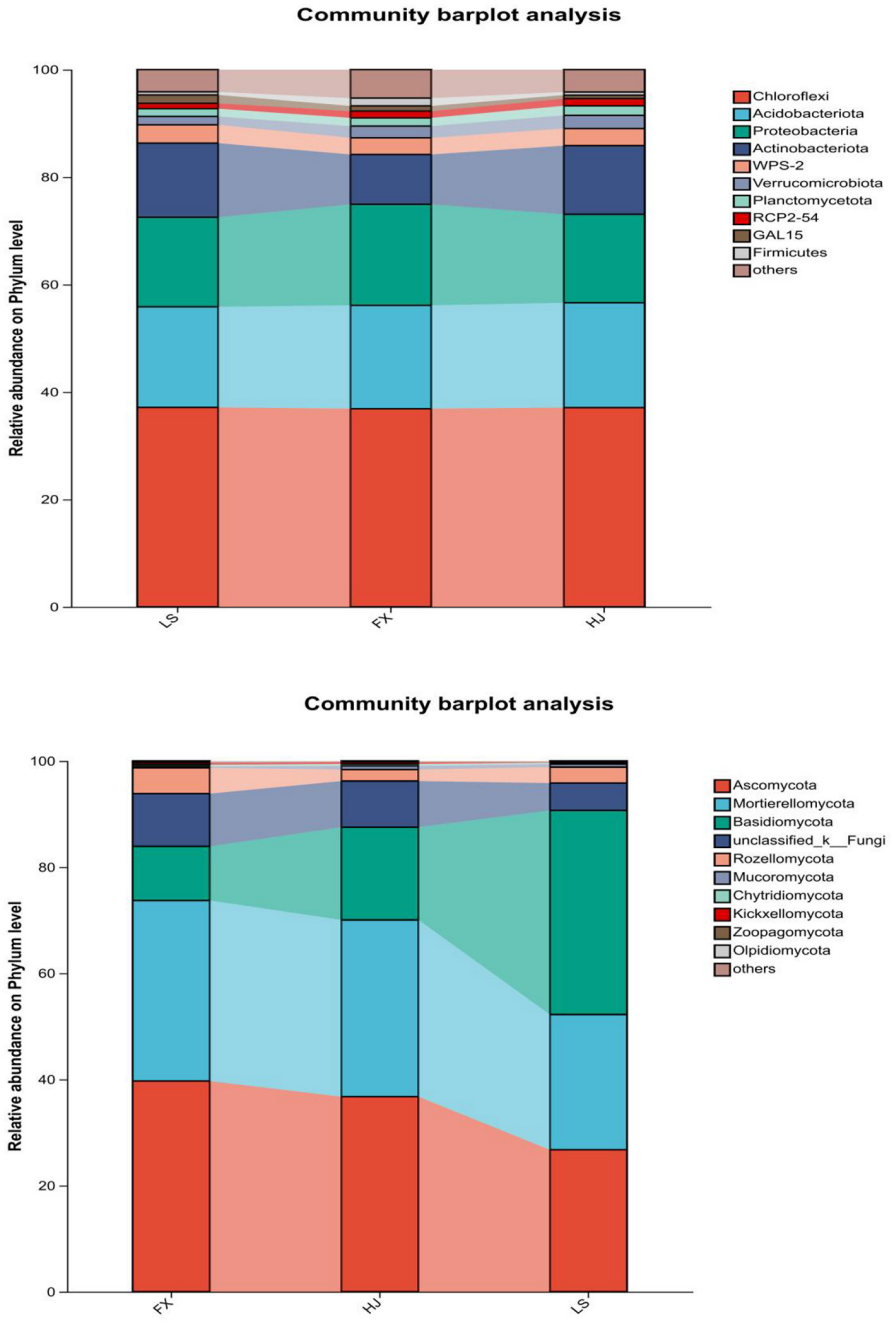
Phylum-Level of Soil Microbial Communities in Different Forest Stand Types (Top:Soil Bacteria; Bottom: Soil Fungi). LS: pure *Cryptomeria fortunei*, FX: pure *Liquidambar formosana*, HJ: mixed forests.

Bar plots at the phylum level visually displayed the abundance of microbial phyla across stand types (Figure 4). The top 10 bacterial phyla in the pure *C. fortunei* forests, pure *L. formosana*, and mixed forests were *Chloroflexi, Acidobacteriota, Proteobacteria, Actinobacteriota, WPS-2, Verrucomicrobiota, Planctomycetota, RCP2-54, GAL15*, and *Firmicutes*. The dominant phyla were *Chloroflexi, Acidobacteriota, Proteobacteria*, and *Actinobacteriota* (Figure 4). Metastats analysis (Table 3) showed: *Actinobacteriota* relative abundance in conifer-broadleaf mixed forests was 37.46% higher than that in pure *L. formosana* forests (*P* < 0.05), *Firmicutes* relative abundance in pure *L. formosana* forests was 158.93% higher than that in pure *C. fortunei* forests and 154.39% higher than that in mixed forests (*P* < 0.05). The top ten fungal phyla across all three stand types were *Ascomycota, Mortierellomycota, Basidiomycota, unclassified_k__Fungi, Rozellomycota, Mucoromycota, Chytridiomycota, Kickxellomycota, Zoopagomycota*, and *Olpidiomycota*, and the dominant phyla were *Ascomycota, Mortierellomycota*, and *Basidiomycota* (Figure 4). Metastats analysis (Table 3) showed that *Ascomycota* relative abundance in *conifer-broadleaf* mixed forests was ~1.37 times higher than that in pure *C. fortunei* forests (*P* < 0.05).

**Table 3:** Relative abundance of soil microbial communities at the phylum level.

Microbial species	Phylum	LS	FX	HJ	*P*_value (FX-LS)	*P*_value (FX-HJ)	*P*_value (LS-HJ)
Soil bacteria	Chloroflexi	37.14%	39.90%	37.11%	*P* > 0.05	*P* > 0.05	*P* > 0.05
	Acidobacteriota	18.73%	19.24%	19.49%	*P* > 0.05	*P* > 0.05	*P* > 0.05
	Proteobacteria	16.66%	18.79%	16.68%	*P* > 0.05	*P* > 0.05	*P* > 0.05
	Actinobacteriota	13.79%	9.29%	12.77%	*P* > 0.05	***P*** **<** **0.05**	*P* > 0.05
	WPS-2	3.40%	3.09%	3.19%	*P* > 0.05	*P* > 0.05	*P* > 0.05
	Verrucomicrobiota	1.56%	2.18%	2.44%	*P* > 0.05	*P* > 0.05	*P* > 0.05
	Planctomycetota	1.44%	1.53%	1.77%	*P* > 0.05	*P* > 0.05	*P* > 0.05
	RCP2-54	1.01%	1.25%	1.34%	*P* > 0.05	*P* > 0.05	*P* > 0.05
	GAL15	1.58%	0.97%	0.67%	*P* > 0.05	*P* > 0.05	*P* > 0.05
	Firmicutes	0.56%	1.45%	0.57%	***P*** **<** **0.05**	***P*** **<** **0.05**	*P* > 0.05
	others	4.14%	5.31%	4.17%			
Soil fungi	Ascomycota	26.74%	39.69%	36.75%	*P* > 0.05	*P* > 0.05	***P*** **<** **0.05**
	Mortierellomycota	25.50%	34.03%	33.31%	*P* > 0.05	*P* > 0.05	*P* > 0.05
	Basidiomycota	38.44%	10.20%	17.48%	*P* > 0.05	*P* > 0.05	*P* > 0.05
	unclassified_k__Fungi	5.16%	9.93%	8.68%	*P* > 0.05	*P* > 0.05	*P* > 0.05
	Rozellomycota	3.00%	4.87%	2.17%	*P* > 0.05	*P* > 0.05	*P* > 0.05
	Mucoromycota	0.56%	0.23%	0.70%	*P* > 0.05	*P* > 0.05	*P* > 0.05
	Chytridiomycota	0.28%	0.33%	0.35%	*P* > 0.05	*P* > 0.05	*P* > 0.05
	Kickxellomycota	0.12%	0.37%	0.35%	*P* > 0.05	*P* > 0.05	*P* > 0.05
	Zoopagomycota	0.09%	0.16%	0.10%	*P* > 0.05	*P* > 0.05	*P* > 0.05
	Olpidiomycota	0.09%	0.06%	0.08%	*P* > 0.05	*P* > 0.05	*P* > 0.05
	others	0.01%	0.12%	0.03%			

LS, pure *Cryptomeria fortunei*; FX, pure *Liquidambar formosana*; HJ, mixed forests. Bold values indicate that this phylum differs significantly between the two stand types.

**Figure 5:**
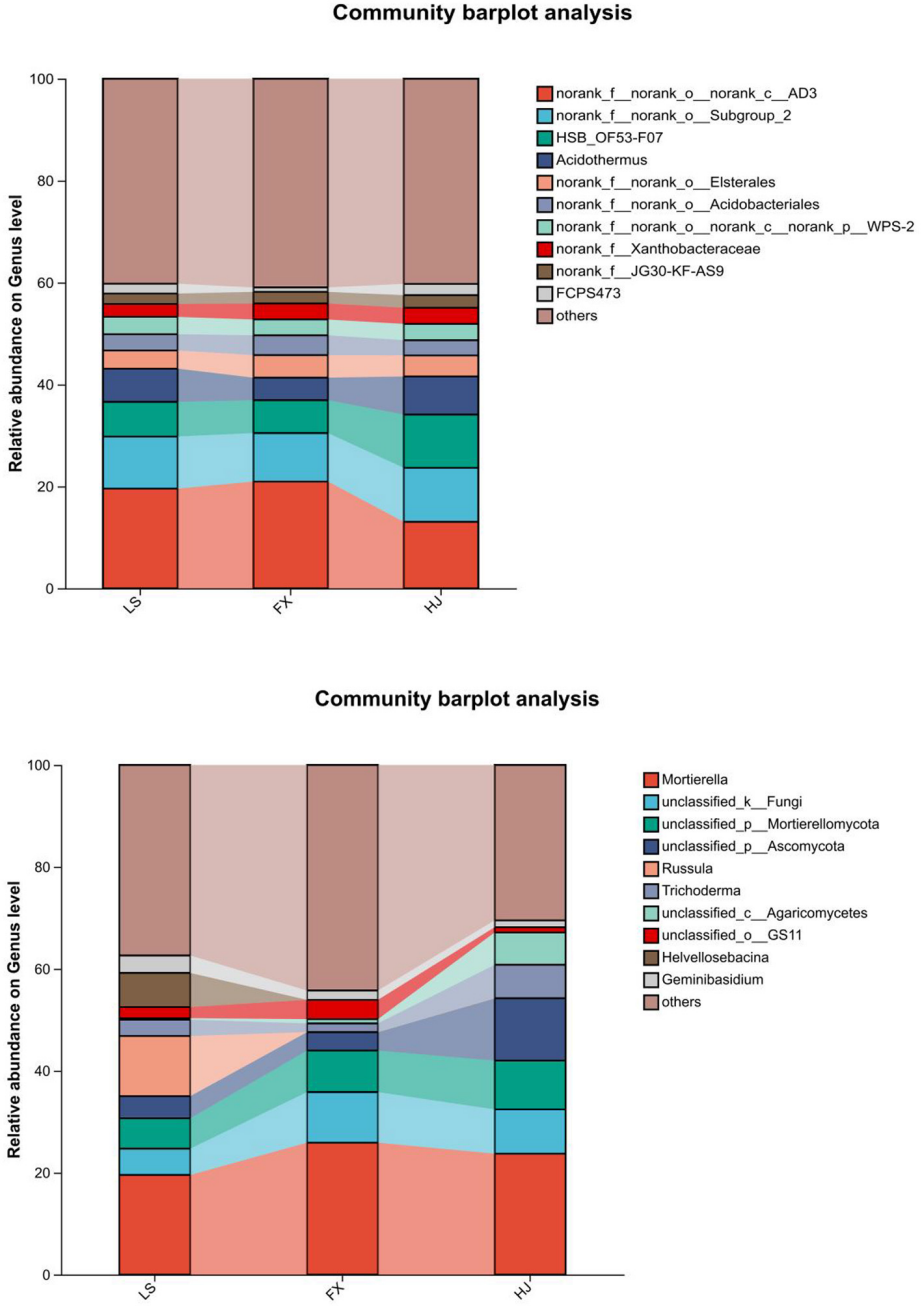
Soil microbial communities at the genus level under different forest types (Top: soil bacteria; Bottom: soil fungi).

The bar plots at the genus level provides an intuitive visualization of the relative abundances of different microbial phyla (Figure 5). The top ten genera in the pure C. fortunei forests, pure L. formosana, and mixed forests were *norank_f__norank_o__norank_c__AD3, norank_f__norank_o__Subgroup*_, *HSB_OF53-F07, Acidothermus, norank_f__norank_o__Elsterales, norank_f__norank_o__Acidobacteriales, norank_f__norank_o__norank_c__norank_p__WPS-2, norank_f__Xanthobacteraceae, norank_f__JG30-KF-AS9, FCPS473* (Figure 4). Metastats analysis of the relative abundances of these ten genera (Table 4) showed no significant differences in their relative abundances among the three forest types. For the soil fungal communities, the top ten genera in relative abundance across the three forest types were *Mortierella, unclassified_k__Fungi, unclassified_p__Mortierellomycota, unclassified_p__Ascomycota, Russula, Trichoderma, unclassified_c__Agaricomycetesunclassified_o__GS11, Helvellosebacina, Geminibasidium* (Table 4). The results showed that the relative abundance of unclassified_p__Ascomycota in the mixed forest was ~2.81 to 3.39 times that in the pure forests (*P* < 0.05). The relative abundances of Trichoderma and unclassified_c__Agaricomycetes in the pure C. fortunei forests were ~0.35 to 1.88 times those in the pure L. formosana forests (*P* < 0.05).

**Table 4:** Relative abundance of soil microbial communities at the genus level.

Microbial species	Phylum	LS	FX	HJ	*P*_value (FX-LS)	*P*_value (FX-HJ)	*P*_value (LS-HJ)
Soil bacteria	norank_f__norank_o__norank_c__AD3	19.57%	20.96%	13.05%	*P* > 0.05	*P* > 0.05	*P* > 0.05
	norank_f__norank_o__Subgroup_2	10.22%	9.51%	10.59%	*P* > 0.05	*P* > 0.05	*P* > 0.05
	HSB_OF53-F07	6.81%	6.46%	10.46%	*P* > 0.05	*P* > 0.05	*P* > 0.05
	Acidothermus	6.52%	4.42%	7.47%	*P* > 0.05	*P* > 0.05	*P* > 0.05
	norank_f__norank_o__Elsterales	3.56%	4.42%	4.13%	*P* > 0.05	*P* > 0.05	*P* > 0.05
	norank_f__norank_o__Acidobacteriales	3.19%	3.89%	2.98%	*P* > 0.05	*P* > 0.05	*P* > 0.05
	norank_f__norank_o__norank_c__norank_ p__WPS-2	3.40%	3.09%	3.19%	*P* > 0.05	*P* > 0.05	*P* > 0.05
	norank_f__Xanthobacteraceae	2.53%	3.14%	3.18%	*P* > 0.05	*P* > 0.05	*P* > 0.05
	norank_f__JG30-KF-AS9	2.05%	2.28%	2.47%	*P* > 0.05	*P* > 0.05	*P* > 0.05
	FCPS473	1.93%	0.88%	2.21%	*P* > 0.05	*P* > 0.05	*P* > 0.05
	others	40.22%	40.95%	40.25%			
Soil fungi	Mortierella	19.55%	25.88%	23.74%	*P* > 0.05	*P* > 0.05	*P* > 0.05
	unclassified_k__Fungi	5.16%	9.93%	8.68%	*P* > 0.05	*P* > 0.05	*P* > 0.05
	unclassified_p__Mortierellomycota	5.95%	8.14%	9.57%	*P* > 0.05	*P* > 0.05	*P* > 0.05
	unclassified_p__Ascomycota	4.35%	3.61%	12.22%	*P* > 0.05	***P*** **<** **0.05**	***P*** **<** **0.05**
	Russula	11.80%	0.02%	0.05%	*-*	*-*	*-*
	Trichoderma	3.21%	1.71%	6.54%	***P*** **<** **0.05**	*P* > 0.05	*P* > 0.05
	unclassified_c__Agaricomycetes	0.29%	0.83%	6.34%	***P*** **<** **0.05**	*P* > 0.05	*P* > 0.05
	unclassified_o__GS11	2.18%	3.79%	1.01%	*P* > 0.05	*P* > 0.05	*P* > 0.05
	Helvellosebacina	6.70%		0.03%	*-*	*-*	*-*
	Geminibasidium	3.42%	1.83%	1.34%	*-*	*-*	*-*
	others	37.39%	44.25%	30.48%			

LS, pure *Cryptomeria fortune*; FX, pure *Liquidambar formosana*; HJ, mixed forests. Bold values indicate that this genus differs significantly between the two stand types.

### 3.3 Heatmap network analysis of soil microbial community composition and environmental variables

**Figure 6:**
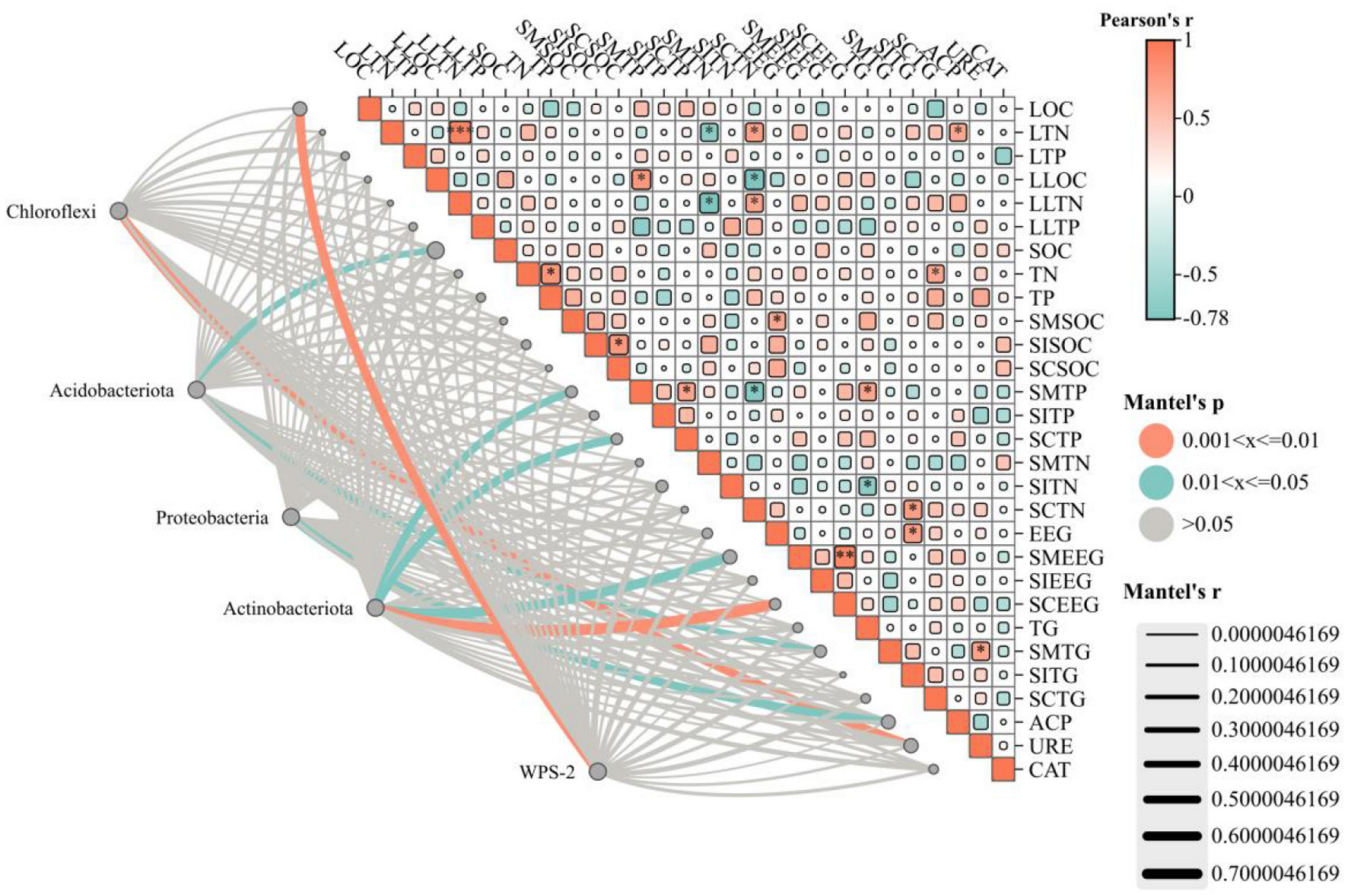
Mantel test network heatmap of phylum-level soil bacterial community and environmental factors. *P < 0.05, **P < 0.01, ***P < 0.001.

**Figure 7:**
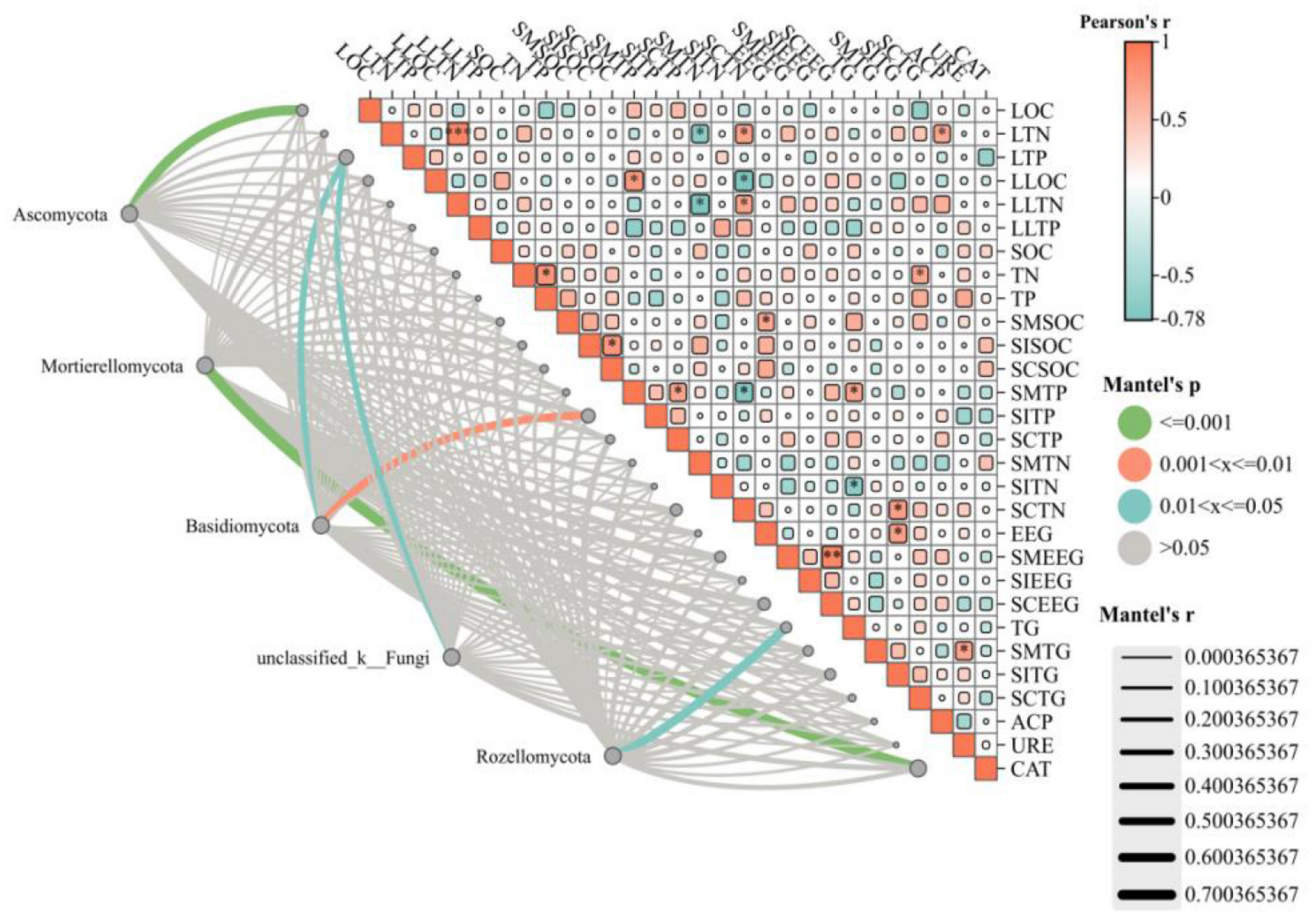
Mantel test network heatmap of phylum-level soil fungal community and environmental factors. *P < 0.05, **P < 0.01, ***P < 0.001.

The Mantel test was performed between environmental variables and the top five phyla in terms of relative abundance, and the results were visualized using heatmap networks. For soil bacteria (Figure 6): *Acidobacteriota* correlated significantly with soil organic carbon and soil macroaggregate total glomalin-related soil protein (*P* < 0.05); *Proteobacteria* correlated significantly with acid soil phosphatase activity (*P* < 0.05); *Actinobacteriota* correlated significantly with soil macroaggregate total phosphorus and silt-clay total particle (*P* < 0.05); *Chloroflexi* correlated extremely significantly with urease activity (*P* < 0.01); *Actinobacteriota* correlated extremely significantly with soil -clay easily-extractable glomalin-related soil protein, (*P* < 0.01); *WPS-2* correlated extremely significantly with leaf organic carbon (*P* < 0.01). For soil fungi (Figure 7), *Basidiomycota* and unclassified_k__fungi correlated significantly with leaf total phosphorus, *Rozellomycota* with total glomalin-related soil protein (*P* < 0.05), *Basidiomycota* correlated extremely significantly with soil macroaggregate total phosphorus (*P* < 0.01), *Ascomycota* correlated extremely significantly with leaf organic carbon (*P* < 0.001), *Mortierellomycota* correlated extremely significantly with catalase activity (*P* < 0.001).

Significant correlations existed among environmental variables: soil easily- extractable glomalin-related soil protein, soil-clay total nitrogen, and catalase exhibited significant correlations with soil intra-microaggregate total glomalin-related soil protein; phosphatase activity with leaf total nitrogen; soil-clay total glomalin-related soil protein, with soil total nitrogen; soil total glomalin-related soil protein with soil macroaggregate total phosphorus; soil easily-extractable glomalin-related soil protein with soil macroaggregate organic carbon; soil-clay total nitrogen with leaf total nitrogen, and litter layer total nitrogen; litter layer organic carbon and soil-clay total phosphorus with soil macroaggregate total phosphorus; soil macroaggregate organic carbon with soil intra-macroaggregate organic carbon; and soil total phosphorus, with soil total nitrogen (*P* < 0.05). Soil clay easily-extractable glomalin-related soil protein exhibited an extremely significant positive correlation with soil microaggregate easily-extractable glomalin-related soil protein (*P* < 0.01), whereas litter layer total nitrogen showed a highly significant positive correlation with litter total nitrogen (*P* < 0.001). Conversely, significant negative correlations were observed between soil total glomalin-related soil protein and soil intra-microaggregate total glomalin-related soil protein, soil macroaggregate total nitrogen and litter layer organic carbon, and soil clay total phosphorus and soil macroaggregate total phosphorus, litter total nitrogen, and litter layer total nitrogen (*P* < 0.05).

### 3.4 Variance partitioning analysis (VPA)

**Figure 8:**
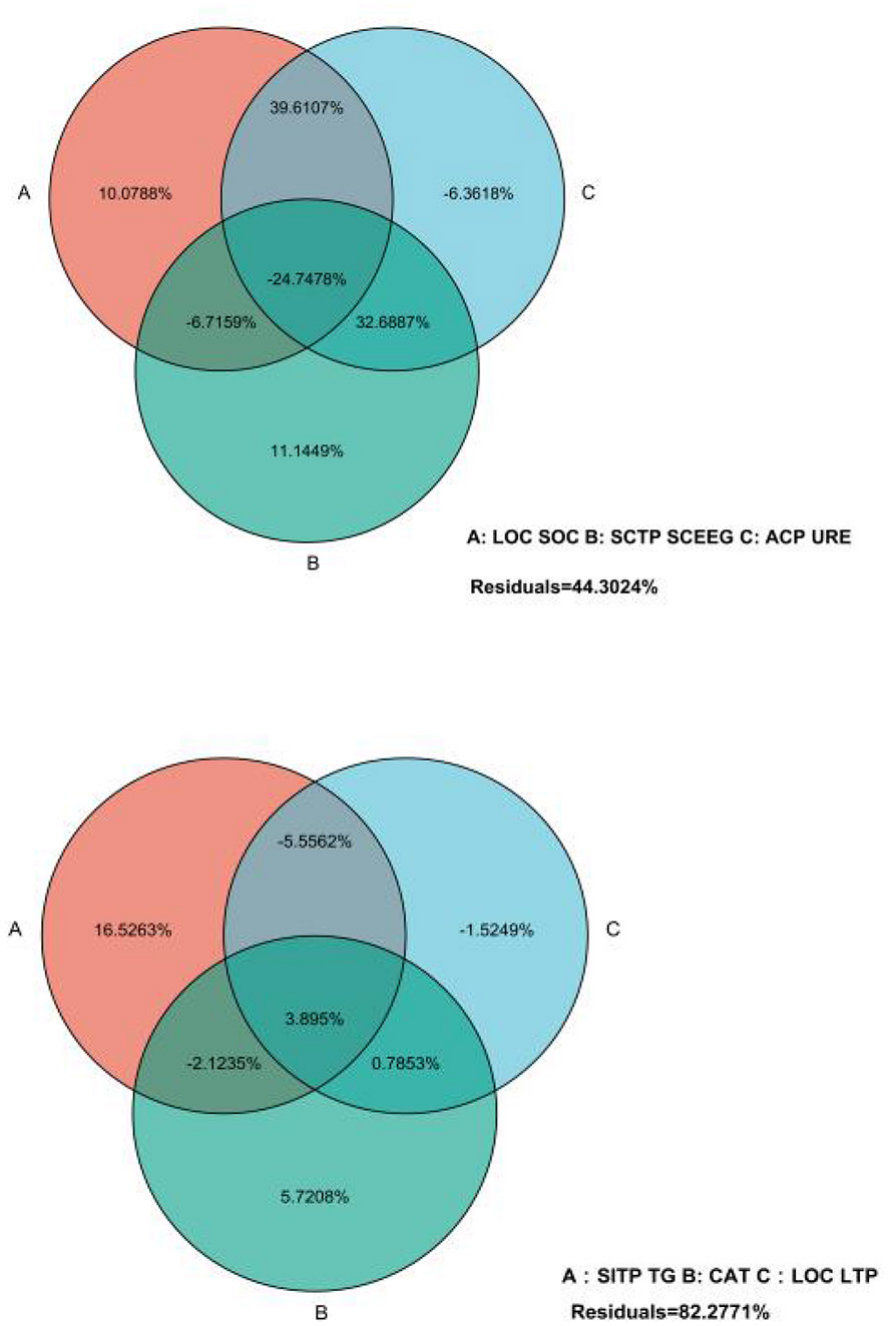
Explanatory power of environmental factors on phylum-level communities (top: soil bacteria; bottom: soil fungi).

Variance Partitioning Analysis (VPA) was conducted using environmental variables that were significantly correlated with soil microbial communities (identified using Mantel tests), which were first screened using Variance Inflation Factor (VIF) analysis to retain variables with minimal multicollinearity. The VPA quantified the contribution of environmental variables to bacterial and fungal community structures. For bacterial communities, the variables significantly associated with composition were grouped as follows: organic carbon (Group A: LOC, SOC), soil nutrients (Group B: SCTP, SMEEG), and enzyme activities (Group C: ACP, URE). Group A individually contributed 10.08%, Group B contributed 11.14%, and the effect of Group C is negligible.. Synergistic effects between enzyme activities and organic carbon accounted for 39.61% of the variation, whereas enzymes and soil nutrients jointly explained 32.69% of variation. The synergistic effect between soil organic carbon and soil nutrients accounts for a relatively small proportion (Figure 8). Consequently, the synergistic effect between enzyme activity and organic carbon exerted the strongest influence on bacterial communities at phylum level, surpassing the impact of individual variables.

Environmental variables that significantly correlated with fungal community composition were grouped into three categories: soil nutrients (Group A: SITP and TG), enzyme activities (Group B: CAT), and leaf nutrients (Group C: LOC and LTP).. Group A contributed 16.53%, Group B contributed 5.72%, and the effect of Group C is negligible. The synergistic effects among groups only account for a small proportion of the total variation (Figure 8). Consequently, soil nutrients exerted the strongest individual influence on fungal communities.

## 4 Discussion

### 4.1 Tree species mixing alters soil microbial community diversity and composition

Karst regions, as typical ecologically fragile areas, exhibit core barren characteristics such as shallow soil, discontinuous soil layers, and an extremely poor capacity for retaining both nutrients and water (Chen et al., 2012). Soil microorganisms constitute the most diverse component of terrestrial biodiversity and play vital roles in ecosystem processes, including carbon-nitrogen cycling and pedogenesis (Guo and Gong, 2024). Therefore, the characteristics of soil microbial communities can reflect the soil quality and ecosystem restoration potential of fragile Karst habitats.

#### 4.1.1 Tree species mixing alters soil microbial community diversity

Soil microbial diversity indicators can be regarded as one of the most promising sensitive biological indicators (Chen et al., 2012). Our α-diversity analysis revealed significantly higher bacterial Smith-Wilson indices in mixed forests than pure *L. formosana* stands (*P* < 0.05, Figure 1), while fungal Coverage indices were significantly lower in mixed forests (*P* < 0.05, Figure 2). The Smith-Wilson index directly reflects the evenness of species distribution within the bacterial community and serves as a core indicator for assessing community stability and functional redundancy. Under the conditions of extreme soil infertility and high resource fluctuation typical of Karst regions, the increased evenness of the bacterial community in mixed forests indicates that the diverse litter inputs and soil microhabitats of coniferous-broadleaf mixed forests with complex community structures can weaken the resource monopoly of single dominant bacterial taxa in Karst habitats, thereby promoting bacterial growth (Zhang et al., 2025b). The Coverage index directly reflects the sequencing coverage of the fungal community. Fungi exhibit strong host specificity and resource specialization (Nan et al., 2023). *Coniferous-broadleaf* mixed forests can break the screening limitation of a single host found in pure forests and enhance the species reserve capacity of fungal communities in fragile Karst habitats. Therefore, a decrease in this value does not signify a decline in fungal diversity, but rather directly reflects the increased complexity of the species pool in mixed forests within Karst habitats. Previous research in Xinjiang, Fujian, Shandong, and Hunan and other regions have found that soil bacterial α-diversity in conifer-broadleaf mixed forests, such as *C. lanceolata* and *Schima superba* was higher than in pure forests, while soil fungal α-diversity in mixed forests was lower than in pure forests, supporting our conclusions (Dong et al., 2021; Guo and Gong, 2024). However, studies on *Phyllostachys edulis, C. lanceolata* mixed forests reported significantly decreased bacterial α-diversity and increased fungal diversity relative to pure stands, contradicting our findings (Jiang et al., 2023). Different stand types can drive the directional reshaping of soil microbial communities through multiple pathways, such as regulating litter input characteristics and root exudate composition (Zhang et al., 2018). Multiple pathways, such as stand structure and soil factor changes, will have an impact on soil microbial community structure (Dong et al., 2021). Owing to differences in soil environmental variables and dominant tree species across the study regions, research outcomes ultimately diverged (Guo et al., 2024).

At the beta diversity level, significant differences were observed in the soil fungal community beta diversity among the three forest types (Figure 3). Compared with bacteria, fungi exhibit a stronger specificity for host plants, root-derived carbon sources, and soil organic matter types (Yang, 2024). This phenomenon may occur because coniferous tree rhizosphere soils in mixed forests exhibit higher organic carbon content and fine root biomass, leading to an altered fungal community structure (Li et al., 2021). Mixing of tree species facilitates the formation of dense root systems and enhances nutrient release, thereby increasing soil fungal activity, further complicating fungal community structure and strengthening their adaptability to environmental changes (Guo and Gong, 2024).

#### 4.1.2 Tree species mixing affects soil microbial community abundance

Research on soil microbial community composition enables an understanding of soil ecosystem health, assessment of soil quality, and provides a basis for ecological restoration (Guo and Gong, 2024). In this study, the relative abundance of *Actinobacteriota* in the soil of coniferous-broadleaf mixed forests was significantly higher, showing a 37.46% increase compared to that in the *Liquidambar formosana* pure forest, while the relative abundance of *Ascomycota* was 1.37 times that of the *Cryptomeria japonica* pure forest (*P* < 0.05, Table 3). Coniferous litter contains substantial amounts of refractory substances such as lignin and cellulose (Chen et al., 2026). *Actinobacteriota* possess the functions of decomposing organic matter, synthesizing antibiotics, and participating in nitrogen cycling (Wang et al., 2026), enabling them to efficiently utilize such scarce resources. It plays a vital role in improving the barren soil ecological environment in karst regions. *Ascomycota* are closely associated with organic matter decomposition and plant interactions (Yang et al., 2026); they primarily utilize labile carbon sources to degrade fresh organic matter and are considered major decomposers of soil organic matter (Wang M. et al., 2025). Compared to non-Karst soils, Karst soils typically have lower phosphorus and nitrogen content (An, 2025). *Coniferous-broadleaf* mixing can improve the soil microbial habitat by increasing the supply of carbon sources, enhancing nitrogen and phosphorus nutrient content, and regulating pH (Deng, 2024), thereby promoting soil quality improvement in the Karst ecosystem. These findings are highly consistent with previous studies: Dong et al. (2021) observed lower relative abundances of *Actinobacteriota* and *Proteobacteria* in mixed forests than in pure *European beech* forests. Conversely, some studies have documented reduced *Ascomycota* but increased *Basidiomycota* or *Glomeromycota* abundance in mixed forests relative to monocultures (Li et al., 2021). Yang et al. (2022b) found no significant differences in fungal abundance between monoculture and mixed communities in Heilongjiang black soils, which diverged from our results. Distinct plant community assemblages formed by different species exert significantly varying effects on soil microbial communities and their associated processes (Gillespie, 2020). Owing to differences in tree species composition, mixed forests alter litter quality, quantity, root biomass, and root exudate chemistry, thereby modifying local microhabitat conditions and consequently influencing soil microbial community composition (Wen et al., 2014).

This study revealed that although the relative abundances of the top ten bacterial genera did not show significant differences among the three forest types, their compositional structure tended to be consistent at the genus level. This suggests that the core soil bacterial communities in different vegetation types in the Karst region possess a certain degree of stability, potentially shaped by the combined effects of environmental filtering at the regional scale. At the fungal level, the *coniferous-broadleaf* mixed forest significantly increased the relative abundance of *unclassified_p__Ascomycota*, which was ~2.81 to 3.39 times higher than that in the pure forests (*P* < 0.05, Table 4). Soil fungi participate in processes such as humus formation and decomposition, ammonification, and nitrification, and play a particularly important role in organic matter transformation in acidic soils (Wang et al., 2022). Mixed forests can improve litter quality and microbial activity, thereby enhancing soil aggregate stability, nutrient availability, and carbon sequestration potential (Fu et al., 2025). This introduces a greater variety of organic materials into the soil, which can provide multiple nutritional sources for fungi, thereby regulating the composition of the soil fungal community (Zhong et al., 2025). In existing research, Zhong et al. (2025), through a comparative analysis of soil fungal community composition and structure between pure *Eucalyptus* forests and mixed forests of Acacia melanoxylon and Eucalyptus, found that the abundances of eight genera (*Penicillium, Xylogone, Rhytidhysteron, Coniosporium, Paraconiothyrium, Lophodermium, Curvularia*, and *Pestalotiopsis*) were significantly higher in the mixed stands, while the opposite trend was observed for *Clonostachys* and *Thelephora* (Zhong et al., 2025). Liang (2024) selected pure *Phyllostachys edulis* forests, *bamboo-forest* mixed zones, and *coniferous-broadleaf* mixed forests as study objects and found that the abundances of 20 fungal genera, including *Mortierella, Tolypocladium, Penicillium*, and *Archaeorhizomyces*, were highest in the *bamboo-forest* mixed zones. Compared with these existing results, the proportion of *unclassified fungi* was slightly higher in this study, suggesting that the mixed environment might provide living conditions for more unknown fungi (Fang et al., 2024).

In conclusion, the *coniferous-broadleaf* mixed forest promoted the optimization of soil microbial community structure such as *Actinobacteriota* and *unclassified_p__Ascomycota*. The core mechanism underlying this improvement lies in the precise regulation of microbial physiological traits and niche adaptability driven by the increased habitat heterogeneity within the mixed forest. These findings not only confirm the pivotal role of forest type in regulating soil microbial communities but also provide a microbiological theoretical foundation for the sustainable management of plantations and the enhancement of soil ecosystem functions.

### 4.2 Synergistic effects of organic carbon and enzymatic activities drive soil bacterial community variation

Soil bacteria play important roles in biogeochemical cycling, maintenance of soil fertility, plant health protection, and regulation of ecological balance (Li et al., 2024). The variance partitioning analysis (VPA) in this study clearly indicated that the synergistic coupling effect of soil organic carbon and enzyme activity explained up to 39.61% of the variation in bacterial community structure (Figure 8).

Previous research confirms that organic carbon content is a key driver of phylum-level bacterial community structure (Wang Q. et al., 2025). As a critical carbon and energy source, organic carbon not only influences bacterial community assembly (Yang et al., 2025) and metabolic functionality (Liu et al., 2025), but also provides essential substrates for bacterial growth (Xu et al., 2018). When soil organic carbon is sufficient, bacteria acquire adequate carbon and energy to sustain their vital activities. Notably, bacteria enhance microbial network complexity to increase soil multifunctionality and exert positive feedbacks on organic carbon accumulation and stabilization (He et al., 2024). This mechanism serves as a crucial microbial pathway for enhancing soil function in the fragile habitats of Karst regions.

Soil enzymes serve as critical indicators for assessing soil microbial activity and functionality (Tiwari et al., 2025), participate in bacterial metabolic processes and nutrient cycling, and function as sensitive proxies for bacterial nutrient demands and soil quality (Trasar-Cepeda et al., 2000; Xu et al., 2017), significantly influencing soil bacterial communities. Concurrently, Soil enzymes play crucial roles in nutrient cycling, with their activities intrinsically linked to soil carbon dynamics (Luo et al., 2017). Changes in enzymatic activities reflect nutrient transformations (Moitinho et al., 2021), whereas soil organic carbon improves soil environments (Wang Q. et al., 2025), providing optimal conditions for enzymatic functions to indirectly regulate their activity. Research indicates that the components of soil active organic carbon are highly responsive to environmental changes and exhibit significant interactive relationships with carbon cycle enzyme activity and microbial community structure (Xu et al., 2021). Moreover, different active carbon components require different enzyme systems for degradation, thus selecting microbial communities with corresponding functional genes (Zhang et al., 2022). This also suggests that future research should explore the changes in microbial community structure and function in combination with organic carbon components and carbon metabolism enzyme activity.

Karst soil is derived from carbonate rocks and is rich in exchangeable calcium ions, which promotes bacterial community diversity and the formation of complex network structures (Wang et al., 2021). This feature also profoundly affects the resource acquisition strategy of microbial communities. Research has shown that the resource limitation patterns faced by soil microorganisms in karst ecosystems are significantly different from those in non karst areas (Chen et al., 2019). In the process of natural vegetation restoration, the phosphorus acquisition strategy of soil microorganisms in karst areas has undergone significant changes (Yang et al., 2021). At the enzyme activity level, karst soils are dominated by carbon demand, while non karst soils are dominated by nitrogen demand (Wang et al., 2021). Our study clarifies that the synergistic explanation rate of these two factors reaches 39.61%. The synergistic regulation of soil bacterial communities by organic carbon and enzyme activity represents a high-degree coupling, at the microbial level, between resource foundation and metabolic catalysis, thereby supplementing the theoretical framework of bacterial community assembly driven by the synergy of organic carbon and enzyme activity in fragile Karst regions.

### 4.3 Soil nutrients as primary drivers of fungal community composition

Soil fungi mediate organic matter decomposition, promote nutrient acquisition, and establish symbiotic associations with plants, thereby fulfilling critical functions in ecosystem stability and phytobiont development, are key functional groups responsible for decomposing organic matter, regulating plant nutrient uptake, and maintaining the stability of the Karst ecosystem (Li et al., 2024). Relative fungal abundance exhibits significant responsiveness to soil nutrient gradients (Fan and Tulli, 2016), which concurrently regulate fungal taxonomic diversity and population dynamics (Zhang et al., 2024), while fungi also play an important role in soil nutrient cycling (Zong et al., 2024), which can improve soil structure and promote soil nutrient cycling, to enhance Karst soil fertility in a reverse direction (Wu et al., 2025). Liu et al. (2024) demonstrated that soil heavy metals, physicochemical properties, and enzymatic activities explained 16.94%, 8.63%, and 7.27% of fungal community variation, respectively, indicating that soil physical and chemical properties, soil heavy metals and soil enzyme activities jointly affected the structure of fungal communities, and soil heavy metals played a leading role. In contrast, our study revealed a higher explanatory power of soil nutrients (total glomalin-related soil protein and microaggregate total phosphorus) over enzymatic activity and foliar nutrients for fungal structural changes (Figure 8). This difference stems from the unique barren attributes of Karst habitats. Glomalin-related soil protein (GRSP), as a protein produced by arbuscular mycorrhizal fungi (AMF), plays a key role in Karst soils, which are characterized by shallow depth and loose structure. The cementing effect of GRSP contributes to the formation of stable soil aggregates, thereby providing a suitable micro-environment that supports the survival and proliferation of soil microorganisms (Zhang et al., 2025a). Furthermore, the decomposition of GRSP can release nutrients that can be used by soil microorganisms, thereby affecting the metabolic activity and diversity of the microbial community (Wu et al., 2024). Phosphorus, which is essential for microbial growth and metabolism, directly modulates microbial activity and community structure through its concentration and bioavailability (Pei et al., 2024). Discrepancies between our VPA outcomes and prior findings stem from variations in research focus, study regions, and selected environmental variables. Consequently, the VPA results of this study differ from those of previous research, primarily due to differences in the study regions' habitats: previous studies were conducted in fertile, non-Karst soils, whereas this study focused on the barren, phosphorus-limited, and structurally fragile ecological zone of Karst regions. In this context, the driving effects of nutrient factors on soil microbial communities were considerably higher than those of other factors. This indicates that the driving factors of soil microbial communities in fragile Karst ecological zones exhibit significant regional specificity. Future research could further expand the range of environmental factors to deepen the understanding of the mechanisms underlying microbial community assembly in Karst habitats.

## 5 Conclusions

This study investigated the effects of tree species mixing on soil microbial community diversity and composition in three stand types, namely pure *C. fortunei*, pure *L. formosana*, and mixed *C. fortunei-L. formosana* forests, within a typical karst region in Southwest China. We further analyzed the influence of leaf, litter, soil nutrients, and enzymatic activities on microbial community assembly. The key findings were as follows: (1) tree species mixing significantly restructured microbial communities. Compared to pure *L. formosana* forests, mixing significantly increased the soil bacterial Smith-Wilson index but decreased the fungal coverage index (*P* < 0.05). Significant divergence in fungal β-diversity occurred among stand types (*P* < 0.05). Mixed forests exhibited 37.46% and 37.43% higher relative abundances of *Actinobacteriota* and *Ascomycota*, respectively, than pure stands. Pure *L. formosana* forests showed 154.39% greater *Firmicutes* abundance than mixed stands (*P* < 0.05).The relative abundance of unclassified_p__Ascomycota in the mixed forest was ~2.81 to 3.39 times that in the pure forests. The relative abundances of Trichoderma and unclassified_c__Agaricomycetes in the pure C. fortunei forests were ~0.35 to 1.88 times those in the pure L. formosana forests (*P* < 0.05). (2) Leaf, soil, and enzymatic activities regulate soil microbial community composition through synergistic and individual effects. Phylum-level microbial composition was significantly correlated with leaf nutrients, soil nutrients, and enzymatic activity. Synergistic effects between organic carbon (soil and leaves) and enzyme activities (soil phosphatase and urease) dominated community variation. For fungi, soil nutrients (total GRSP and total microaggregate phosphorus) had the strongest individual influence.

## 6 Limitations of the study

This study was conducted in Zhazuo Forest Farm of Guizhou Province. Restricted by research duration and study area, only three stand types were selected for plot establishment, with three replicates for each plot. Based on this setup, we explored the effects of stand types on soil microbial communities. Given the relatively small sample size in this research, statistical power may be low, the results have certain limitations and can only serve as a theoretical reference for ecological restoration in karst regions. In follow-up work, we will expand the research scope, set up more sampling plots, and further refine the research conclusions.

## Data Availability

The original contributions presented in the study are included in the article/supplementary material, further inquiries can be directed to the corresponding authors.The data presented in the study are deposited in the NCBI Sequence Read Archive (SRA) repository, accession number is PRJNA1482995.
